# Assessing the effect of risk factors on rates of obstetric transfusion over time using two methodological approaches

**DOI:** 10.1186/s12874-018-0595-6

**Published:** 2018-11-16

**Authors:** Edward Jegasothy, Jillian Patterson, Deborah Randall, Tanya A. Nippita, Judy M. Simpson, David O. Irving, Jane B. Ford

**Affiliations:** 1 Clinical and Population Perinatal Health Research, Kolling Institute, Northern Sydney Local Health District, St Leonards, NSW Australia; 2 Biostatistics Training Program, New South Wales Ministry of Health, North Sydney, NSW Australia; 3 Sydney Medical School Northern, University of Sydney, St Leonards, NSW Australia; 4 Department of Obstetrics and Gynaecology, Royal North Shore Hospital, St Leonards, NSW Australia; 5 Sydney School of Public Health, University of Sydney, Sydney, NSW Australia; 6 Australian Red Cross Blood Service, Sydney, NSW Australia

**Keywords:** Blood transfusions, Obstetrics, Trends

## Abstract

**Background:**

While red blood cell transfusion rates have declined in most Australian medical specialties, obstetric transfusion rates have instead been increasing. Obstetric transfusions are mostly linked to postpartum haemorrhage, the rates of which have also increased over time. This study used two methodological approaches to investigate recent trends in obstetric transfusion in New South Wales (NSW) and the extent to which this was influenced by changing maternal and pregnancy characteristics.

**Methods:**

Linked birth and hospital records were used to examine rates of red blood cell transfusion in the postpartum period for mothers giving birth in NSW hospitals from 2005 to 2015. Logistic regression models were run to examine the contribution of maternal and pregnancy risk factors to changing rates of transfusion. Risk factors were divided into “pre-pregnancy” and “pregnancy related”. Crude and adjusted estimates of the effect of year of birth on obstetric transfusion rates were compared to assess the effect of risk factors on rates over time using two approaches. The first compared actual and predicted odds ratios of transfusion for each year. The second compared the observed increase in transfusion rate with that predicted after controlling for the risk factors.

**Results:**

Among 935,659 births, the rate of obstetric transfusion rose from 13 per 1000 births in 2005 to 17 in 2011, and remained stable until 2015. From 2005 to 2015, postpartum haemorrhage increased from 74 to 114 per 1000 births. Compared with the rate in 2005, the available maternal and pregnancy characteristics only partially explained the change in rate of transfusion by 2015 (Method 1, crude odds ratio 1.39 (95% CI 1.25, 1.56); adjusted odds ratio 1.29 (95% CI 1.15, 1.45)). After adjustment for maternal and pregnancy characteristics, obstetric transfusion incidence was predicted to increase by 10.3%, but a 38.7% increase was observed (Method 2).

**Conclusion:**

Rates of obstetric transfusion have stabilised after a period of increase. The trend could not be fully explained by measured maternal and pregnancy characteristics with either of the two approaches. Further investigation of rates and maternal and clinical risk factors will help to inform and improve obstetric blood product use.

**Electronic supplementary material:**

The online version of this article (10.1186/s12874-018-0595-6) contains supplementary material, which is available to authorized users.

## Background

There has been a global movement to reduce the usage of red blood cell transfusions and focus on patient blood management to prevent avoidable blood loss [1]. In Australia, the overall usage of red blood cells has been decreasing [2]. This decrease has been attributed to programmes designed to standardise the use of blood products, such as the implementation of patient blood management guidelines. However, rates of obstetric transfusion were still increasing up to 2010 [3]. These transfusions, given to mothers around childbirth, are primarily given as treatment following postpartum haemorrhage (PPH) [3] and make up 3.8% of all red blood cells issued [4].

In 2015, the National Blood Authority of Australia released the Patient Blood Management Guidelines module for Obstetrics and Maternity [5]. These guidelines detailed evidence- and experience-based best practice for blood management specifically within the obstetric setting, including transfusion, detection and management of anaemia, use of recombinant activated factor VII, tranexamic acid, cell salvage and interventional radiology. Prior to this, there were no specific guidelines for obstetric transfusions in place in Australia. However, evidence for patient blood management was already in development and guidelines for Critical Bleeding and Massive Transfusion were published in 2011 [6].

The use of red blood cells in the obstetric setting is influenced by patient indications as well as clinical practice [7]. There are numerous risk factors contributing to the risk of obstetric transfusion. These include demographics and baseline health of the mother (such as maternal age, parity, previous caesarean section) as well as conditions and treatments occurring during the antenatal and peripartum period (induction of labour, instrumental delivery) [3], and these risk factors are changing over time. PPH rates have also increased [8]. While transfusion rates have been explored up until 2010, more recent trends in obstetric transfusion, and the extent to which maternal, pregnancy and birth factors may be influencing the changing rate of transfusion, are unknown.

This study aimed to assess the recent trend in red blood cell transfusion rates for mothers giving birth in hospitals in NSW, Australia, and, using two methodological approaches, the extent to which this trend can be explained by available maternal, pregnancy and birth characteristics.

## Methods

### Study population

The study population comprised women delivering live or stillborn infants of at least 20 weeks gestation in New South Wales (NSW) hospitals from July 2005 to June 2015. NSW is the most populous state in Australia with a population of 7.6 million residents in 2015 and accounts for approximately one third of all Australian births [9].

### Data sources

Data on maternal and birth characteristics were from the Perinatal Data Collection (‘birth data’), a record of all births occurring in NSW, reported by the attending midwife or doctor. The Admitted Patient Data Collection (‘hospital data’) provided hospital inpatient admission records including diagnoses and procedures coded with the 10th revision of the International Classification of Diseases, Australian Modification (ICD10-AM) and the Australian Classification of Health Interventions (ACHI), respectively. These two datasets underwent probabilistic linkage by the NSW Centre for Health Record Linkage, with an estimated false positive and false negative linkage rate of less than 5 per 1000 records [10]. Datasets were provided to the researchers with identifying fields removed.

### Outcome

The outcome of interest was the administration of a red blood cell transfusion during the birth admission and in postnatal hospital admissions up to six weeks after the birth (ACHI 13706–01 or 13,706–02), referred to herein as an ‘obstetric transfusion’. This would have included a small number of transfusions that were administered in the birth admission but prior to delivery.

### Risk factors

We identified potential maternal and pregnancy characteristics known to contribute to the risk of an obstetric transfusion through literature review and clinical experience [3, 8, 11–16]. We then assessed which of these were reliably available in the data sources. The factors were categorised as pre-pregnancy, or pregnancy and birth (Additional file 1: Table S1).

Maternal demographics, and pregnancy, labour and birth characteristics were obtained from the birth data. Acute and chronic conditions were ascertained from the hospital data, by searching diagnosis codes of the antenatal, birth and postpartum admissions. Anaemia diagnoses were identified from antenatal records only. In vitro fertilisation and intracytoplasmic sperm injection (‘assisted reproductive technology’) were identified if the mother was admitted to hospital with these diagnosis or procedure codes in the twelve months prior to the birth. As hospital data were only available from July 2001, previous postpartum haemorrhage and obstetric transfusion was derived from a four-year look-back period in the linked data prior to the birth. A previous validation study, which compared medical records to hospital discharge data, found that red blood cell transfusion (sensitivity 83.1%, positive predictive value 98.8%) and PPH (sensitivity 73.8%, positive predictive value 83.9%) are well reported in hospital data, though there is some under-ascertainment [17]. The use of linked hospital and births data decreases the risk of misclassification of certain characteristics compared with each of the data sources individually [18, 19].

Maternal socioeconomic status (SES) was derived from each mother’s area of residence using the Index of Relative Socioeconomic Advantage and Disadvantage (IRSAD) from the Socioeconomic Indexes for Areas package produced by the Australian Bureau of Statistics [20]. Each area of residence was assigned a quintile of IRSAD based on population distribution.

### Statistical analysis

Descriptive analyses of the outcome and risk factors were conducted by plotting rates of each by year. We used logistic regression to estimate the crude effects on the outcome of each of the risk factors as well as year of birth. Cochran-Armitage tests were also performed to assess the trend in rates of obstetric transfusions, PPH and each of the covariates, by year, over the study period.

We used two approaches to assess the extent to which maternal, pregnancy and birth factors could explain the temporal trends in obstetric transfusion rate. The first method assessed the trend in transfusions over time that is not explained by risk factors, and the second demonstrated the expected trend given the risk factors. Both methods used predictive logistic regression models with obstetric transfusion as the outcome while controlling for risk factors. Using these models, with maternal and pregnancy risk factors as predictors, we predicted the rates of red blood cell transfusion over the study period. If changes in maternal and pregnancy risk factors were sufficient to explain the increasing transfusion rate, this would be reflected in the close fit of the models.

In the first approach, crude odds ratios for year of birth were compared with adjusted odds ratios for year of birth from models: (a) adjusted for pre-pregnancy risk factors, and (b) adjusted for all pre-pregnancy, pregnancy and birth risk factors, which also include pregnancy complications. Pre-pregnancy factors include maternal demographics, reproductive history and existing comorbidities. The full model adjusted for all of the available pre-pregnancy, current pregnancy and birth factors. The degree to which the adjusted odds ratios moved closer to 1 compared with the crude odds ratios indicated how much of the temporal trend was explained by the risk factors.

The second method compared the change in observed rate of obstetric transfusion by year with the predicted rate of transfusion by year from a model with (a) only pre-pregnancy risk factors, and (b) all pre-pregnancy, pregnancy and birth risk factors. These models did not include year of birth as a covariate and included data from all years of the study. The relative increase in the observed rate by year compared with 2005 was calculated by dividing the observed rate in each year by the rate in 2005, thus indicating the proportional change in obstetric transfusions over time. Similarly, the predicted rate-ratios by year showed the relative expected change in rate of transfusion due to the changing distribution of risk factors over time. Both approaches assumed a consistent relationship between each of the risk factors and the outcome over the study period.

Covariates were excluded if they could not be consistently ascertained over the study period. We also examined the trends in PPH, but because it is on the causal pathway between many of the risk factors and the outcome PPH was not included as a risk factor in the analyses. Statistical analysis was performed using SAS, version 9.3 (SAS Institute, Cary, North Carolina, United States of America).

## Results

**Fig. 1:**
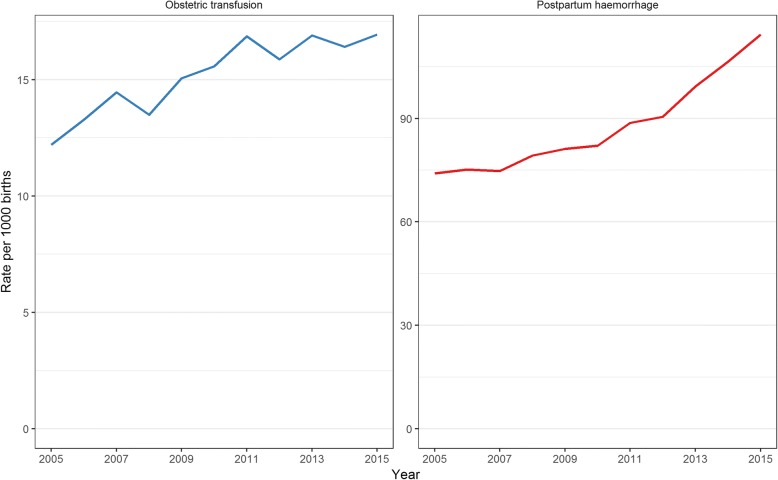
Rates of obstetric transfusion (left) and postpartum haemorrhage (right) in New South Wales, 2005–2015

There were 939,470 births in NSW hospitals between July 2005 and June 2015. Of these, 3811 (0.4%) births were excluded due to missing values for one or more risk factors. Among the remaining 935,659 births included in the study, mothers were found to have received an obstetric red blood cell transfusion in 14,275 (15 per 1000 births) births. This rate increased by 38.7% from 13 per 1000 births in 2005 to a peak of 17 per 1000 births in 2011 (increasing trend, *p* < 0.001), but then remained mostly stable until 2015, remaining at 17 per 1000 births for each year apart from a drop to 16 per 1000 births in 2012 (Fig. 1). The rate of postpartum haemorrhage followed a similar trend to that of obstetric transfusion (Fig. 1) up to 2011, but continued to increase after 2011. Overall, the PPH rate increased by 54% from 74 in 2005 to 114 per 1000 births in 2015 (*p* < 0.0001). Of mothers receiving obstetric transfusions, 76.7% were found to have had PPH (Table 1). The proportions of mothers with several risk factors were found to have an increasing trend over the study period including mothers over 40 years of age, mothers born overseas, previous obstetric transfusions, diabetes, forceps delivery and caesarean sections, both with and without labour (all *p* < 0.0001) (Table 1).

**Table 1:** Maternal and pregnancy characteristics of women giving birth in New South Wales hospitals, 2005–2015, for those with and without obstetric transfusion and by year

			Obstetric transfusion	No obstetric transfusion	2005–06	2014–15	Trend test^b^
			N (per 1000 births)		Prevalence^c^ (per 1000 births)		*p*
	Total		*N* = 14,275	*N* = 921,384	*N* = 88,707	N = 92,875	
Pre-pregnancy risk factors	Maternal age						
	< 20 years		740 (51.8)	30,356 (32.9)	38.2	26.7	< 0.0001
	20–24 years		2221 (155.6)	122,355 (132.8)	142.8	125.0	< 0.0001
	25–29 years		3751 (262.8)	249,994 (271.3)	270.6	272.5	0.001
	30–34 years		4256 (298.1)	305,705 (331.8)	337.8	347.0	< 0.0001
	35–39 years		2562 (179.5)	174,513 (189.4)	175.0	184.4	0.57
	40+ years		745 (52.2)	38,461 (41.7)	35.7	44.4	< 0.0001
	Smoker		1919 (134.4)	105,603 (114.6)	138.2	93.4	< 0.0001
	Parity						
	1st		7417 (519.6)	394,021 (427.6)	415.4	438.9	< 0.0001
	2nd		3520 (246.6)	309,134 (335.5)	334.5	340.6	0.88
	3rd		1763 (123.5)	136,140 (147.8)	155.6	139.0	< 0.0001
	4th		791 (55.4)	48,837 (53.0)	57.1	48.2	< 0.0001
	5+		784 (54.9)	33,252 (36.1)	37.3	33.4	< 0.0001
	Multiple birth		658 (46.1)	13,214 (14.3)	16.0	14.6	0.0047
	Australian born		9231 (646.7)	621,316 (674.3)	719.8	635.6	< 0.0001
	Assisted reproductive technology		571 (40.0)	24,078 (26.1)	20.9	28.2	< 0.0001
	Previous obstetric transfusion		491 (34.4)	7346 (8.0)	4.4	11.5	< 0.0001
	Previous PPH		1349 (94.5)	45,633 (49.5)	31.9	64.0	< 0.0001
	Previous caesarean or uterine scar		2185 (153.1)	145,638 (158.1)	143.7	165.2	< 0.0001
	Pregestational diabetes		105 (5.5)	5040 (5.5)	5.1	7.5	< 0.0001
	Pregestational hypertension		242 (11.1)	10,191 (11.1)	12.5	10.8	0.01
	Any chronic condition^a^		847 (59.3)	22,005 (23.9)	34.7	26.4	< 0.0001
	Blood/platelet disorder		1642 (115.0)	9118 (9.9)	12.5	13.5	0.078
	Morbid obesity		157 (11.0)	5148 (5.6)	2.5	9.5	< 0.0001
	Private hospital/insurance		3033 (212.5)	307,650 (333.9)	326.3	323.6	< 0.0001
	Quintile of socioeconomic status						
	Lowest quintile		3448 (241.5)	193,810 (210.3)	218.4	209.3	< 0.0001
	Second quintile		3246 (227.4)	184,444 (200.2)	199.5	208.2	< 0.0001
	Middle quintile		2907 (203.6)	177,755 (192.9)	190.8	195.4	0.0001
	Fourth quintile		2528 (177.1)	177,314 (192.4)	186.2	195.9	< 0.0001
	Highest quintile		1987 (139.2)	178,375 (193.6)	196.0	183.9	< 0.0001
	Area of residence unknown		159 (11.1)	9686 (10.5)	9.0	7.2	< 0.0001
Pregnancy and birth risk factors	Anaemia in pregnancy		197 (13.8)	2949 (3.2)	1.9	8.0	< 0.0001
	Antepartum haemorrhage		727 (50.9)	21,823 (23.7)	24.0	26.7	< 0.0001
	Postpartum haemorrhage		10,947 (766.9)	70,633 (76.7)	73.8	111.6	< 0.0001
	Placenta praevia		1076 (75.4)	9277 (10.1)	10.0	11.1	0.13
	Placental abruption		545 (38.2)	4143 (4.5)	4.8	5.5	0.0073
	Morbidly adherent placenta		724 (50.7)	1675 (1.8)	2.3	3.1	0.0498
	Retained placental tissue		105 (7.4)	4316 (4.7)	5.7	3.3	< 0.0001
	Uterine rupture		129 (9.0)	345 (0.4)	0.6	0.5	0.18
	Uterine fibroid		79 (5.5)	1302 (1.4)	2.8	1.2	< 0.0001
	Pregnancy diabetes		1097 (76.8)	62,109 (67.4)	48.6	119.4	< 0.0001
	Pregnancy hypertension		2051 (143.7)	72,579 (78.8)	85.8	79.1	< 0.0001
	Gestational age						
	20–32 weeks		835 (58.5)	13,687 (14.9)	15.3	16.3	0.68
	33–36 weeks		1404 (98.4)	47,079 (51.1)	49.5	54.7	< 0.0001
	37–41 weeks		11,874 (831.8)	853,244 (926.0)	917.8	925.2	< 0.0001
	42+ weeks		162 (11.3)	7374 (8.0)	17.4	3.8	< 0.0001
	Large for gestational age		2150 (849.4)	92,783 (100.7)	97.1	97.3	< 0.0001
	Mode of birth						
	Normal vaginal delivery		6115 (428.4)	536,899 (582.7)	609.3	562.0	< 0.0001
	Forceps		1457 (102.1)	35,638 (38.7)	31.6	47.5	< 0.0001
	Vacuum		1394 (97.7)	65,074 (70.6)	69.7	65.5	< 0.0001
	Vaginal breech		110 (7.7)	3338 (3.6)	3.6	3.8	0.83
	Caesarean with labour		2540 (177.9)	115,015 (124.8)	116.9	126.4	< 0.0001
	Caesarean without labour		2659 (186.3)	165,420 (179.5)	168.8	194.7	< 0.0001
	Onset of labour						
	Spontaneous		6752 (473.0)	508,947 (552.4)	580.7	505.3	< 0.0001
	Induced		4864 (340.7)	247,007 (268.1)	250.5	300.0	< 0.0001
	No labour		2659 (186.3)	165,430 (179.5)	168.8	194.7	< 0.0001
	3rd/4th degree perineal tear		969 (67.9)	18,820 (20.4)	18.3	23.8	< 0.0001
	Episiotomy		2950 (206.7)	115,758 (125.6)	115.0	142.6	< 0.0001
	Cervical laceration		248 (17.4)	554 (0.6)	0.7	1.1	0.0013

^a^Chronic conditions include, psychiatric, renal, cardiovascular, autoimmune, respiratory and thyroid illnesses

^b^Cochran-Armitage trend test for prevalence of risk factor over years 2005 to 2015

^c^Prevalence of risk factor among births 1 July 2005 to 30 June 2006 and 1July 2014 to 30 June 2015

In unadjusted analysis, women who received obstetric transfusion were more likely to be giving birth to their first child, giving birth pre-term (< 37 weeks gestation) or giving birth to multiples compared with those who did not have a transfusion (Table 2). Those who had a previous PPH or obstetric transfusion, an inherited or acquired haematological or platelet disorder, a third or fourth degree perineal tear, an episiotomy, an antepartum haemorrhage, a large-for-gestational-age infant, any placental abnormality, higher parity (5+), induced labour or hypertension, were at increased risk of transfusion. Women from lower socioeconomic status areas were more likely to receive a transfusion than those from higher socioeconomic status areas. There was no difference in risk of transfusion between mothers who had a previous caesarean or previous uterine surgery and those who did not (Table 2).

**Table 2:** Crude and adjusted odds ratios of obstetric transfusion in women giving birth in New South Wales hospitals, 2005–2015 (Method 1)

	Crude odds ratios (95% CI)	Adjusted odds ratios (95% CI)	
		Pre-pregnancy	Full model
Year of birth			
2005	Ref	Ref	Ref
2006	1.09 (0.98,1.21)	1.08 (0.98,1.20)	1.09 (0.98,1.21)
2007	1.19 (1.07,1.31)	1.19 (1.07,1.32)	1.16 (1.05,1.29)
2008	1.10 (1.00,1.22)	1.12 (1.01,1.23)	1.09 (0.98,1.21)
2009	1.24 (1.12,1.36)	1.27 (1.15,1.40)	1.26 (1.13,1.39)
2010	1.28 (1.16,1.41)	1.30 (1.18,1.44)	1.29 (1.16,1.43)
2011	1.38 (1.25,1.53)	1.40 (1.27,1.55)	1.37 (1.24,1.52)
2012	1.30 (1.18,1.44)	1.29 (1.17,1.43)	1.26 (1.14,1.39)
2013	1.39 (1.26,1.53)	1.36 (1.23,1.50)	1.34 (1.21,1.48)
2014	1.35 (1.22,1.49)	1.30 (1.17,1.43)	1.27 (1.14,1.40)
2015	1.39 (1.25,1.56)	1.34 (1.20,1.50)	1.29 (1.15,1.45)
Maternal age			
< 20 years	1.34 (1.23,1.46)	1.20 (1.10,1.31)	1.33 (1.22,1.45)
20–24 years	Ref	Ref	Ref
25–29 years	0.83 (0.78,0.87)	0.93 (0.88,0.98)	0.86 (0.81,0.91)
30–34 years	0.77 (0.73,0.81)	0.99 (0.93,1.04)	0.87 (0.82,0.92)
35–39 years	0.81 (0.76,0.86)	1.08 (1.01,1.15)	0.90 (0.84,0.96)
40+ years	1.07 (0.98,1.16)	1.31 (1.20,1.44)	1.06 (0.97,1.17)
Smoke	1.20 (1.14,1.26)	1.02 (0.97,1.08)	1.01 (0.96,1.07)
Parity			
1st	Ref	Ref	Ref
2nd	0.60 (0.58,0.63)	0.54 (0.51,0.56)	0.77 (0.73,0.81)
3rd	0.69 (0.65,0.72)	0.58 (0.55,0.62)	0.87 (0.82,0.92)
4th	0.86 (0.80,0.93)	0.67 (0.62,0.72)	0.98 (0.90,1.07)
5+	1.25 (1.16,1.35)	0.86 (0.79,0.93)	1.21 (1.10,1.32)
Multiple	3.32 (3.07,3.60)	2.94 (2.71,3.20)	2.50 (2.28,2.74)
Australian born	0.88 (0.85,0.91)	0.91 (0.88,0.94)	0.89 (0.86,0.92)
Assisted reproductive technology	1.55 (1.43,1.69)	1.66 (1.52,1.82)	1.47 (1.33,1.61)
Previous obstetric transfusion	4.66 (4.17,5.20)	3.00 (2.64,3.42)	2.73 (2.38,3.13)
Previous postpartum haemorrhage	1.98 (1.85,2.12)	1.88 (1.73,2.04)	1.82 (1.68,1.98)
Uterine scar	0.96 (0.92,1.01)	1.27 (1.20,1.33)	1.08 (1.02,1.16)
Pregestational diabetes	1.35 (1.11,1.64)	0.97 (0.79,1.19)	0.78 (0.63,0.95)
Pregestational hypertension	1.54 (1.36,1.75)	1.22 (1.06,1.39)	0.99 (0.86,1.13)
Chronic condition	2.58 (2.40,2.77)	1.74 (1.61,1.88)	1.64 (1.51,1.77)
Blood disorder	13.01 (12.31,13.75)	11.31 (10.68,11.98)	10.37 (9.76,11.02)
Morbid obesity	1.98 (1.69,2.32)	1.41 (1.20,1.67)	1.26 (1.07,1.50)
Private insurance	0.54 (0.52,0.56)	0.56 (0.53,0.58)	0.56 (0.54,0.59)
Quintile of socioeconomic status			
Most disadvantaged quintile	Ref	Ref	Ref
Second quintile	0.99 (0.94,1.04)	1.03 (0.98,1.08)	1.02 (0.97,1.07)
Middle quintile	0.92 (0.87,0.97)	1.01 (0.95,1.06)	0.95 (0.90,1.00)
Fourth quintile	0.80 (0.76,0.84)	0.88 (0.83,0.93)	0.85 (0.80,0.90)
Most advantaged quintile	0.63 (0.59,0.66)	0.75 (0.70,0.79)	0.72 (0.68,0.77)
Area of residence unknown	0.92 (0.79,1.08)	0.91 (0.78,1.07)	0.96 (0.81,1.13)
Pregnancy anaemia	4.36 (3.77,5.04)	–	1.20 (1.02,1.42)
Antepartum haemorrhage	2.21 (2.05,2.39)	–	1.22 (1.12,1.32)
Placenta praevia	8.02 (7.51,8.56)	–	6.89 (6.35,7.49)
Placental abruption	8.79 (8.03,9.63)	–	5.60 (5.04,6.22)
Morbidly adherent placenta	29.34 (26.84,32.06)	–	22.08 (19.87,24.53)
Retained placental tissue	1.57 (1.30,1.91)	–	0.88 (0.71,1.09)
Uterine rupture	24.35 (19.88,29.83)	–	18.63 (14.76,23.52)
Uterine fibroids	3.94 (3.14,4.95)	–	3.21 (2.49,4.13)
Gestational diabetes	1.15 (1.08,1.23)	–	0.90 (0.84,0.97)
Gestational hypertension	1.96 (1.87,2.06)	–	1.46 (1.38,1.54)
Gestational age			
20–32 weeks	4.39 (4.08,4.71)	–	2.09 (1.91,2.29)
33–36 weeks	2.14 (2.03,2.27)	–	1.28 (1.20,1.37)
37–41 weeks	Ref	–	Ref
42+ weeks	1.58 (1.35,1.85)	–	1.38 (1.17,1.62)
Large for gestational age	1.58 (1.51,1.66)	–	1.62 (1.54,1.70)
Mode of delivery			
Normal vaginal	Ref	–	Ref
Caesarean with labour	1.94 (1.85,2.03)	–	1.60 (1.52,1.69)
Caesarean without labour	1.41 (1.35,1.48)	–	1.06 (0.99,1.13)
Forceps	3.59 (3.39,3.80)	–	2.19 (2.03,2.36)
Vacuum	1.88 (1.77,1.99)	–	1.56 (1.46,1.67)
Vaginal breech	2.89 (2.39,3.50)	–	1.29 (1.04,1.59)
Induced delivery	1.41 (1.36,1.46)	–	1.26 (1.21,1.31)
3rd of 4th degree perineal tear	3.49 (3.27,3.73)	–	2.82 (2.62,3.03)
Episiotomy	1.81 (1.74,1.89)	–	1.53 (1.44,1.62)
Cervical laceration	29.40 (25.28,34.18)	–	24.75 (20.96,29.22)

From the models which included year, if the risk factors could account for the change in rates of transfusion over time, then the odds ratios for each year in the adjusted model would be closer to 1 than the crude odds ratios. Controlling for pre-pregnancy characteristics did not change the effect estimates for year of birth meaningfully when compared with the crude estimates from 2006 to 2010, but slightly larger changes were seen in later years; crude odds ratio (OR) 1.35 (95% confidence interval (CI) 1.22, 1.49) to adjusted OR 1.30 (95% CI 1.17, 1.43) in 2014 and crude OR 1.39 (95% CI 1.25,1.56) to adjusted OR 1.34 (95% CI 1.20,1.50) in 2015 (Table 2). Additional inclusion of pregnancy and birth characteristics saw a further reduction in odds ratios for each of the years. The crude OR for obstetric transfusion comparing 2015 with 2005 was 1.39 (95% CI 1.25, 1.56). This dropped slightly to 1.34 (95% CI 1.20, 1.50) after adjusting for the maternal and pre-pregnancy factors, and further to 1.29 (95% CI 1.15, 1.45) when controlling for all available covariates (Table 2).

**Fig. 2:**
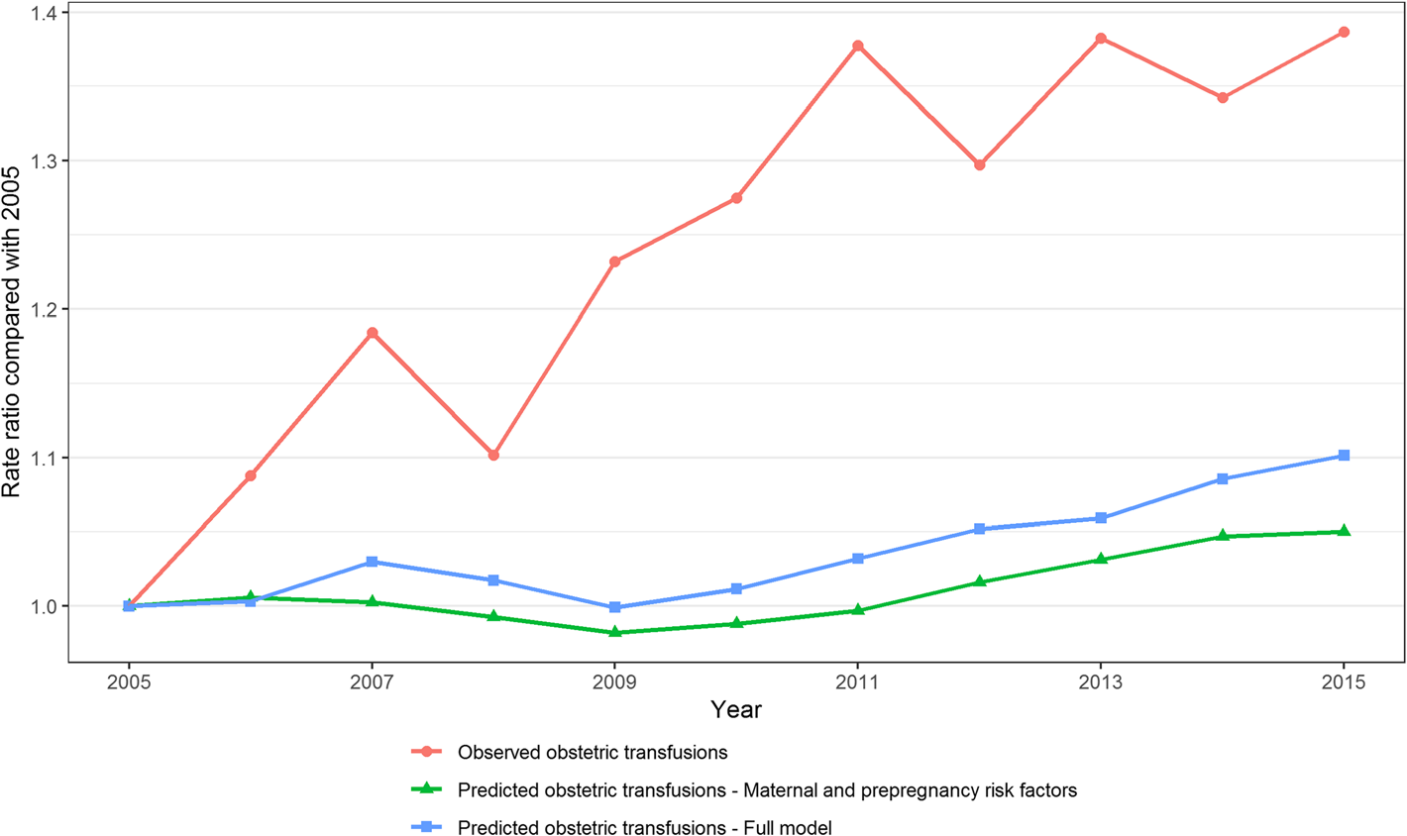
Observed and predicted obstetric transfusion rate-ratios comparing each year with 2005 (Method 2)

The same pattern is shown graphically in Fig. 2 which presents the predicted and observed rate ratios relative to 2005. Considering only pre-pregnancy risk factors, the rate of obstetric transfusion was only expected to rise by 4.1% from 2005 to 2015 (Fig. 2). When including pregnancy and birth risk factors, the expected increase in obstetric transfusion was predicted to be 10.3%. However, there was a 38.7% observed increase in rate over the same period (Fig. 2). The period during which the largest increases in transfusion rate were predicted, in each model, was 2011 to 2015. This is the same period in which the observed rate did not increase.

## Discussion

We found that, among births in NSW hospitals, the rate of obstetric transfusions increased by 38.7% between 2005 and 2011, from 13 to 17 per 1000 births, but remained relatively stable from 2011 to 2015. The two methodological approaches used both revealed that the change in rate of obstetric transfusions could only be partly explained by the available known maternal and pregnancy risk factors. Method 1, comparing crude and adjusted odds ratios, showed that the year effect could not be removed by adjusting for maternal and pregnancy factors. In Method 2, pre-pregnancy risk factors predicted a small, 4.1%, increase in the obstetric transfusion rate. Inclusion of pregnancy and birth factors predicted a larger, 10.3% increase in the rate, but not to the extent that was observed. The majority (76.7%) of obstetric transfusions in the study period were found to coincide with a PPH diagnosis, the rate of which increased throughout the study period, from 74 to 114 per 1000 births.

The increase in PPH and transfusion rates observed up to 2011 is consistent with previous studies in NSW [3, 8]. In the USA, the rate of transfusion also increased from 1998 to 2011, however PPH rates remained stable over the same period. [21] The PPH rate increased in the Netherlands from 40 per 1000 births in 2003 to 66/1000 in 2011 but appeared to plateau from 2011 to 64/1000 in 2013 [22] and in Canada the rate increased by 21% from 51/1000 in 2003–2004 to 6.1/1000 births in 2009–2010 [15].

This is the first study to report a largely stable rate of obstetric transfusions in NSW since 2011. Given that transfusion is often used as a marker of severity of haemorrhage, the stable transfusion rate in the context of increasing PPH is an encouraging sign. However, the obstetric transfusion rate in NSW is still higher than observed in the United States of America where there were 7 transfusions per 1000 births between 1998 and 2011 [21].

We found that pre-pregnancy characteristics, while being important risk factors for transfusion, did not contribute greatly to the change in obstetric transfusion rates over time. Accounting for all the pre-pregnancy, and pregnancy and birth factors that were ascertained, explained a larger degree of the change in transfusion rate but still only predicted a quarter of the increase observed from 2005 to 2015. Interestingly, the largest predicted increases in transfusion, with each model, were seen between 2010 and 2015 when the observed rate remained stable. This means that the rate of obstetric transfusion did not increase during this period despite an overall increase in maternal risk factors for obstetric transfusion. Our findings indicate that changes in rates of obstetric transfusion are largely influenced by other unmeasured factors which may include clinical decision making, policy changes, and the unpredictable nature of PPH as it is understood that many women who experience PPH do not possess any identifiable risk factors [23]. The diagnosis of PPH, based on an estimation of blood loss, and the decision to transfuse are both dependent on perceptions and experience of the attending clinicians as well as the hospital practice and blood supply [7, 11]. Furthermore, the combination of factors prompting intervention, relationships between risk factors or changing management of factors such as induction of labour may have played a role in obstetric transfusion trends.

While the new Patient Blood Management guidelines for Obstetrics and Maternity were only released in 2015 [5], the general movement away from transfusion-based management to patient-based management had been in progress for a number of years prior. The first module of the guidelines for patient blood management in the context of Critical Bleeding and Massive Transfusion was released in 2011 and included some recommendations for maternal care in the area of massive transfusion [6]. This is the same year that the rates of transfusion in the study appeared to peak. The guidelines included treatment paths to reduce transfusion for any critically bleeding or trauma hospital patient and reflected the movement towards reduced use of blood products to reduce unnecessary risk associated with this exposure. Specific recommendations regarding pharmacotherapy include the use of recombinant activated Factor VII [6].

Alongside new guidelines, other treatments for PPH and anaemia have been approved by the Australian Therapeutic Goods Authority (ATGA) and came into clinical practice during the study period, which may have contributed to the stable obstetric transfusion rate. These include tranexamic acid, recombinant activated Factor VII and ferric carboxymaltose (Ferinject). Intravenous tranexamic acid, an antifibrinolytic agent, was approved by ATGA in 2010 to reduce blood loss in cardiac and orthopaedic surgical patients [24]. However, studies since 2001 suggest that tranexamic acid has a role in reducing bleeding and risk of transfusion in postpartum haemorrhage [25–28], such that WHO updated its guideline for management of postpartum haemorrhage to include tranexamic acid in 2012 [29, 30]. Furthermore, the publication of the WOMBAT trial in 2017 and subsequent updates in PPH guidelines for tranexamic acid to be part of standard management, will potentially lead to a reduction in PPH and transfusion rates in the future [31]. Recombinant activated Factor VII is used in Australia to prevent, slow or stop bleeding [32, 33]. Ferric carboxymaltose, introduced in Australia in 2011, is an intravenous infusion given to treat iron deficiency that has advantages over previously available iron treatments including reduced side effects and easier administration [34, 35]. The increased use of such treatments could potentially have had an impact on the rate of transfusions administered to mothers in response to anaemia and haemorrhage; however, this information is not collected in routinely collected birth or hospital data. Further research into specific blood management treatments and variation in practice by hospital may better explain the changes in trends in transfusion over time and point to the potential for improvements in practice to encourage further safe reductions in the use of blood products.

The main strengths of our study were the size and completeness of the dataset as well as the high-quality linked data from both the perinatal and hospital data. All births in NSW hospitals were included in the study with only 0.4% being excluded from the analysis due to missing data. This allowed us to investigate rare risk factors and treatments. While PPH and transfusion codes have been found to be reliable, changes in ascertainment may play a small part in changes in reported rates. This study also used two approaches to answer the study question which provided similar results, strengthening the conclusions. There were, however, some key covariates that are not well reported in either dataset, including body mass index, a change in which over time may have contributed to the changing rate of transfusions. The hospital data did not include laboratory results such as haemoglobin, which may have helped indicate the appropriateness of red blood cell transfusion. We were also limited by our inability to include prior obstetric history of mothers before the availability of linked data in July 2001.While we have taken into account risk factors related to transfusion, particularly in the predicted versus observed analysis, we have assumed the relationship between these factors and risk of transfusion has not changed over time. However, induction of labour (for example) may be more or less likely to result in PPH and transfusion at the beginning compared to the end of our study period.

## Conclusion

The rate of obstetric transfusion among NSW hospital-based births increased over the period of 2005 to 2011 but has since stabilised through to 2015. Meanwhile, the rate of postpartum haemorrhage continued to rise from 2005 through to 2015. The changes in rate of transfusion could only be partly explained by changes in maternal and pregnancy risk factors using each of the methods employed in this study. Further monitoring and investigation, especially following the implementation of specific patient blood management guidelines for obstetrics in 2015, will give deeper insight into these trends to assist policy makers and clinicians to improve best-practice administration of blood products in an obstetric setting.

## Additional file

Additional file 1: **Table S1.** Maternal and pregnancy risk factors for obstetric transfusion and sources. (DOCX 18 kb)

## Abbreviations

ACHI: Australian Classification of Health Interventions
CI: Confidence interval
ICD10-AM: 10th revision of the International Classification of Diseases, Australian Modification
IRSAD: Index of Relative Socioeconomic Advantage and Disadvantage
NSW: New South Wales
PPH: Postpartum haemorrhage
SES: Socioeconomic status
