# Supplementary material for: Assessing the effect of risk factors on rates of obstetric transfusion over time using two methodological approaches
Source: BMC Med Res Methodol. 2018 Nov 16;18:139. doi: 10.1186/s12874-018-0595-6 (PMC6240252; doi:10.1186/s12874-018-0595-6)
Supplement: Supplementary file 1 — Table S1. Maternal and pregnancy risk factors for obstetric transfusion and sources. (DOCX 18 kb) [file 12874_2018_595_MOESM1_ESM.docx]

## Supplementary material

Supplementary Table 1. Maternal and pregnancy risk factors for obstetric transfusion and sources

| Variable | Data source | ICD10/ACHI codes | Values | Pre-pregnancy model | Full model |
| --- | --- | --- | --- | --- | --- |
| Year of birth | Births data |  | 2005-2015 | x | x |
| Maternal age (years) | Births data |  | <20; 20-24; 25-29; 30-34; 35-39; 40+ | x | x |
| Smoke | Births data |  | Yes; No | x | x |
| Parity | Births data |  | 1st; 2nd; 3rd; 4th; 5+ | x | x |
| Multiple | Births data |  | Yes; No | x | x |
| Australian born | Hospital data |  | Yes; No | x | x |
| Assisted reproductive technology | Hospital data | Z31.2; 13251-00; 13218-00; 13218-01 | Yes; No | x | x |
| Previous obstetric transfusion | Hospital data | 13706-01; 13706-02 | Yes; No | x | x |
| Previous postpartum haemorrhage | Hospital data | O72 | Yes; No | x | x |
| Previous caesarean or uterine scar | Births/Hospital data | O34.2 | Yes; No | x | x |
| Diabetes | Births/Hospital data | O24; E10-14 | Yes; No | x (pre-pregnancy only) | x |
| Hypertension | Births/Hospital data | O10-16 | Yes; No | x (pre-pregnancy only) | x |
| Chronic condition | Hospital data | Various cardiac, renal, thyroid, respiratory, psychiatric, autoimmune conditions | Yes; No | x | x |
| Blood disorder | Hospital data | D56-69; D72-77 | Yes; No | x | x |
| Morbid obesity | Hospital data | E65-66 | Yes; No | x | x |
| Pregnancy anaemia | Hospital data | O99.0 | Yes; No |  | x |
| Antepartum haemorrhage | Hospital data | O46 | Yes; No |  | x |
| Postpartum haemorrhage | Hospital data | O72 | Yes; No |  |  |
| Placenta praevia | Hospital data | O44.0-1 | Yes; No |  | x |
| Placental abruption | Hospital data | O45 | Yes; No |  | x |
| Morbidly adherent placenta | Hospital data | O43.2 | Yes; No |  | x |
| Retained placental tissue | Hospital data | O73 | Yes; No |  | x |
| Uterine rupture | Hospital data | O71.0-1 | Yes; No |  | x |
| Uterine fibroids | Hospital data | D25 | Yes; No |  | x |
| Gestational age | Births data |  | Yes; No |  | x |
| Large for gestational age | Births data |  | Yes; No |  | x |
| Mode of delivery | Births data |  | Normal vaginal; caesarean with labour; caesarean without labour; forceps; vacuum; vaginal breech |  | x |
| Induced delivery | Births data |  | Yes; No |  | x |
| 3rd of 4th degree perineal tear | Births/Hospital data | O70.2-3; 90472-00; 16573-00 | Yes; No |  | x |
| Episiotomy | Births/Hospital data | 90472-00 | Yes; No |  | x |
| Cervical laceration | Hospital data | O71.3 | Yes; No |  | x |
| Private insurance | Hospital data |  | Yes; No | x | x |
| Quintile of socioeconomic status | Hospital data |  | 1-5; unknown | x | x |
